# Clinical significance of integrin αV and β superfamily members and focal adhesion kinase activity in oral squamous cell carcinoma: a retrospective observational study

**DOI:** 10.3389/pore.2024.1611571

**Published:** 2024-01-18

**Authors:** Shigeru Sakurai, Yasutaka Ishida, Tomoaki Shintani, Sachiko Yamasaki, Kensaku Matsui, Tomoaki Hamana, Tadayoshi Nobumoto, Souichi Yanamoto, Yasutaka Hayashido

**Affiliations:** ^1^ Department of Oral Oncology, Graduate School of Biomedical and Health Science, Hiroshima University, Hiroshima, Japan; ^2^ Center of Oral Clinical Examination, Hiroshima University Hospital, Hiroshima, Japan

**Keywords:** prognosis, oral squamous cell carcinoma, integrin β superfamily members, FAK signaling pathway, integrin αv

## Abstract

**Objectives:** Integrins are heterodimeric transmembrane plasma membrane proteins composed of α- and β-chains. They bind to extracellular matrix (ECM) and cytoskeletal proteins as ECM protein receptors. Upon ECM protein binding, integrins activate focal adhesion kinase (FAK) and transduce various signals. Despite their importance, integrin and FAK expression in oral squamous cell carcinoma (OSCC) tissue and the prognosis of patients with OSCC remains elusive.

**Methods:** In a retrospective observational study, we immunohistochemically evaluated integrin αV, β1, β3, β5, β6, FAK, and phosphorylated-FAK (pFAK) expressions as prognostic predictors in 96 patients with OSCC. Patients were classified as positive or negative based on staining intensity, and clinicopathologic characteristics and survival rates of the two groups were compared. The association between above integrin-related proteins and PD-1 or PD-L1 in OSCC tissues was investigated.

**Results:** We observed immunohistochemical integrin αV, β1, β6, β8, and FAK expressions in the cell membrane and cytoplasm but not integrin β3 and β5 in the OSCC tissues. pFAK was expressed in the cytoplasm of OSCC cells. The overall survival rate significantly decreased in pFAK-positive OSCC patients compared to the negative group, and cervical lymph node metastasis significantly increased in integrin β8-positive patients with OSCC (*p* < 0.05). No association between integrin-related proteins and PD-1 or PD-L1 in OSCC tissues was observed.

**Conclusion:** Our results indicate that pFAK and integrin β8 are prognostic factors for OSCC. Therefore, pFAK- and integrin β8-targeting new oral cancer diagnostic and therapeutic methods hold a promising potential.

## Introduction

Cancer invasion and metastasis are regulated by both the cancer cell characteristics and their interactions with the host, including surrounding stromal cells and components [1–6]. Extracellular matrix (ECM) proteins, major stromal components, are responsible for maintaining tissue and organ architecture and, like humoral factors such as growth factors and cytokines, they exert various biological activities. These activities occur upon integrin binding on the cell membrane and are crucial for cancer invasion and metastasis as regulators of cancer cell growth, proteolytic enzyme production, cell adhesion, and motility [7–11].

Integrins are α and β chain-containing heterodimeric transmembrane proteins with 18 and 8 reported α and β chain types, respectively, forming 24 types of heterodimers [12–14]. The extracellular integrin domain bind ECM proteins while the intracellular binds various cytoskeletal proteins (e.g., talin, α-actinin, and actin filaments) as ECM protein receptors [15–17]. Furthermore, integrins activate focal adhesion kinase (FAK) bound to their intracellular domain β-chain by binding extracellular substrate proteins, thereby transducing various signals [18–22]. Therefore, integrins are not only adhesion molecules between cellular and ECM proteins but also pivotal for organogenesis and tissue differentiation by regulating cell morphology, motility, proliferation, differentiation, and other signal transduction processes [23–27].

The expression of various integrin molecules reportedly correlates with tumor invasion, metastasis, and prognosis in various malignant tumors [8, 10, 28–30]. In particular, the integrin αV family is closely associated with malignant tumor growth and progression [31–35]. We have previously described that integrin αV-high expressing oral squamous cell carcinoma (OSCC) cell proliferation and invasive potential is promoted by type I collagen [36]. Integrin αVβ6 has been studied in more detail in OSCC. Ramos et al. reported that αVβ6 integrin is upregulated in oral cancer, being central to cancer cell migration as well as to reduced fibronectin matrix deposition and assembly [37]. The authors also demonstrated that integrin αVβ6 regulates epithelial-to-mesenchymal transition in oral cancer [38]. Increased αVβ6 integrin and metalloproteinase-3 expression-related alterations in collagen fibers are associated with unfavorable clinical prognostic factors and reduced survival in OSCC [39]. However, integrin β chain expression, function, or prognostic impact in OSCC remain less understood apart from the β6 chain-related aspects [40, 41].

This study aimed at clarifying the relationship between the expression of integrin αV and its counterparts β1, β3, β5, β6, and β8 in OSCC tissues and OSCC progression and prognosis. In addition, we also performed the immunohistochemical assessment of FAK and phosphorylated-FAK (pFAK) expression, closely involved in integrin-mediated signal transduction. Finally, we investigated the relationship between the expression of the abovementioned molecules and that of PD-1 or PD-L1.

## Materials and methods

### Patients and specimens

Of the 210 patients with OSCC treated by surgical procedures at the Department of Oral and Maxillofacial Surgery, Hiroshima University Hospital between January 2001 and September 2013, we enrolled in this study with 96 available biopsy specimens for immunohistochemical stainings. The inclusion criteria were as follows: archived prior-to-treatment biopsy sample availability, clinical data, and OSCC pathological diagnosis. Living patients underwent at least 6 months of follow-up care. The exclusion criteria included a history of surgery on the primary tumor, chemotherapy, and radiotherapy. We obtained formalin-fixed and paraffin-embedded (FFPE) tissue samples prior to the treatments. The data collected from the medical chart of the patients included age, sex, site of lesion, treatment details, and disease classification on the TNM classification of the International Union for Cancer Control (UICC), 8th Ed [42]. T and N statuses were assessed using depth of invasion (DOI) and extra nodal extension (ENE) based on histopathological examination of specimens. DOI was microscopically measured by an oral surgeon, whereas ENE was classified as ENE (+) in the presence of soft tissue invasion with strong adherence to underlying muscle/adjacent tissue or clinical signs of nerve invasion. Stages 3 and 4 were defined as advanced.

### Immunohistochemical staining

The specific immunohistochemical staining methods we applied in this study have been described previously [43–45]. Supplementary Table S1 summarizes the list of primary and secondary antibodies used in this study.

For staining evaluation, we divided the specimens into three groups according to the staining intensity as follows: 1) no staining at all, 2) weak staining, and 3) strong staining, with group 1 being considered negative while 2 and 3 being judged positive.

### Ethical considerations

This retrospective observational study was approved by the Research Ethics Board of Hiroshima University (Approval no. epidemiology 2023-0025). The study protocol was posted on websites. Participants could opt out of the study if they did not wish to provide consent. The need for informed consent was waived.

### Statistical analysis

We analyzed the correlations between integrin family, FAK, or pFAK expression and each clinicopathological factor using Chi-squared or Student’s t-test. We calculated the survival curves using the Kaplan-Meier method and the log-rank test to assess significant differences in overall survival (OS) rates between integrin family, FAK, and pFAK-negative and positive groups. We analyzed the relation between the expression of the abovementioned integrin-related proteins and that of PD-1 or PD-L1 using chi-square tests. Univariate analysis using the chi-square test was performed to compare clinicopathological variables between groups with and without lymph node metastases. Next, we applied a logistic regression analysis to estimate the odds ratio and 95% confidence interval (CI) of lymph node metastasis that could serve as a risk factor, including age, the positive expressions of integrin β8, or FAK. We evaluated the associations between the examined integrin, FAK, and pFAK expressions as well as the OS rate using the Cox proportional hazards model-estimated hazard ratios with a 95% CI. We performed Cox regression analyses incorporating an interaction term between pFAK and selected patient characteristics (age and stage) to determine if these variables impeded the effect of pFAK expression on the OS rate. We considered differences significant at risk rates of <5% and performed all statistical analyses using the JMP 17 statistical software (SAS Institute Inc., Cary, NC, United States).

## Results

### Baseline characteristics

Of the 96 patients, 56 and 40 were males and females, respectively, with a mean age of 66 years. By site of onset, 10, 15, 36, 4, and 31 patients had carcinoma of the floor of the mouth, maxillary gingival carcinoma, mandibular gingival carcinoma, buccal mucosa carcinoma, and tongue carcinoma, respectively. N0–3 cervical lymph node metastases could be observed in 51, 17, 28, and 45 patients, respectively. Finally, 10, 31, 16, and 39 patients were in Stages I–IV, respectively (Table 1).

**TABLE 1:** OSCC patients and tumor characteristics.

Characteristics	*N*	%
OSCC patients	96	
Age (Mean ± SD)	66.2 ± 12.9	
Sex		
Male	56	58.3
Female	40	41.7
Localization		
Tongue	31	32.3
Maxillary gingiva	15	15.6
Mandibular gingiva	36	37.5
Buccal mucosa	4	4.2
Plantar of the mouth	10	10.4
UICC stage		
Ⅰ	10	10.4
Ⅱ	31	42.4
Ⅲ	16	9.1
Ⅳ	39	30.3
Cervical lymph node metastasis		
Negative	51	53.1
Positive	45	46.9

OSCC, oral squamous cell carcinoma; SD, standard deviation; UICC, international union for cancer control.

### Expression of integrins, FAK, and pFAK in OSCC

**FIGURE 1:**
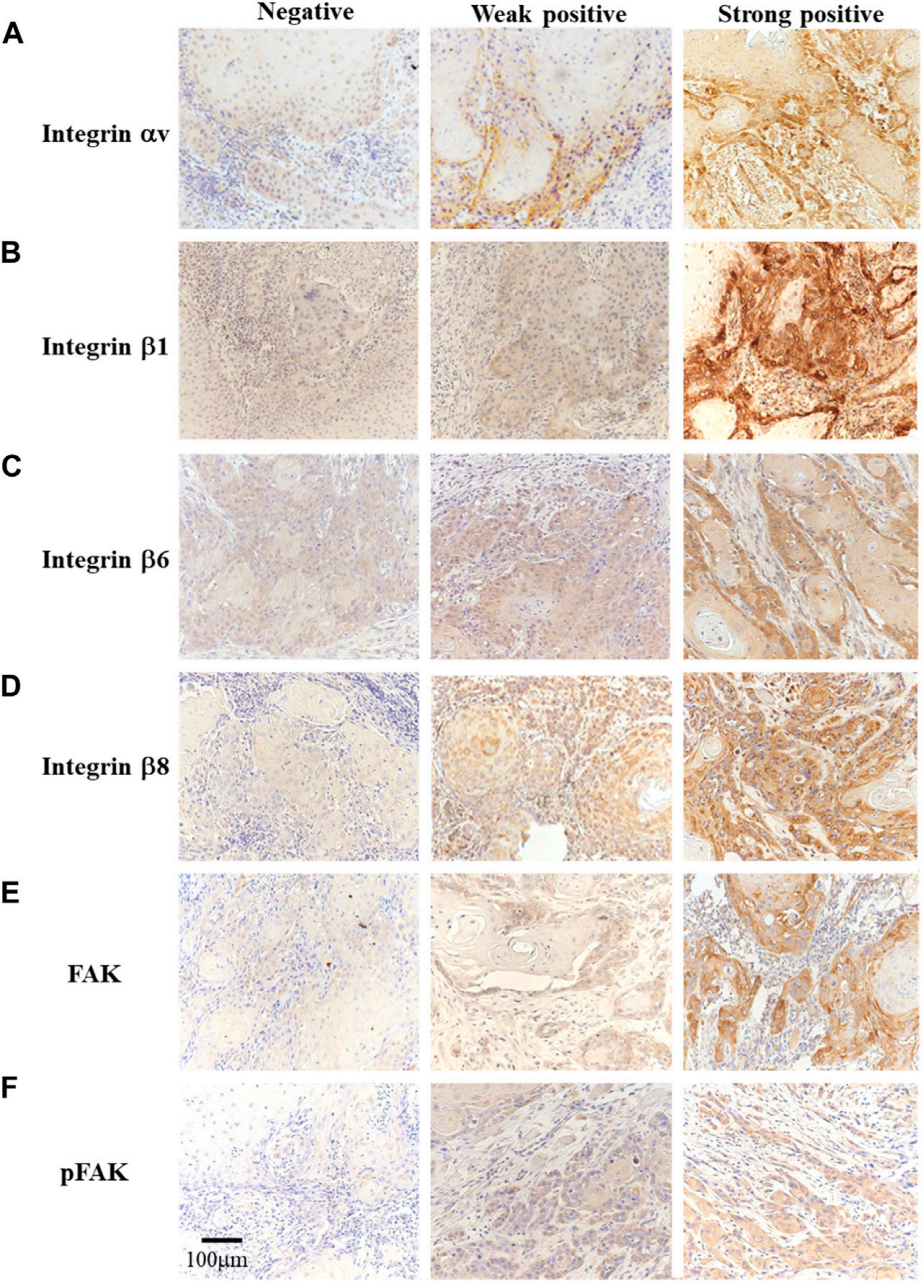
Immunohistochemistry for integrin αV, β1, β6, β8, FAK, and pFAK identification [**(A–F)**, original magnification ×200]. The participants were divided into three groups based on the immunostaining results: negative (left), weak positive (center), and strong positive (right).

First, we immunohistochemically investigated integrin αV and β superfamily member expressions and FAK activity in OSCC. Integrin αV, β1, β6, and β8 expressions localized in the cell membrane and cytoplasm of OSCC cells in the weak- and strong-positive groups, but not in the negative group (Figure 1) while no expression of integrin β3 and β5 was found in FFPE tissue from OSCC patients in this study. FAK was expressed in the OSCC cell membrane and cytoplasm while pFAK in the cytoplasm in the weak- and strong-positive groups, but not in the negative group (Figure 1).

### Comparison of clinicopathological variables between integrin-, FAK-, and pFAK-negative and positive groups

Next, we compared the clinical variables between the positive and negative groups for each integrin-related proteins. The FAK-positive group exhibited significantly more advanced stages than the negative group (Table 2, *p* < 0.05). We observed no significant differences in other clinical variables between the integrin αV, β1, β6, FAK, and pFAK-positive and negative groups.

**TABLE 2:** Comparison of clinical variables between positive and negative groups for integrin or FAK expression.

Characteristics	Integrin αv			Integrin β1			Integrin β6			Integrin β8		
	Negative	Positive	*p*-value	Negative	Positive	*p*-value	Negative	Positive	*p*-value	Negative	Positive	*p*-value
Age (mean ± SD)^a^	66.8 ± 11.1	65.2 ± 13.0	0.55	66.7 ± 13.3	64.1 ± 11.9	0.34	67.4 ± 11.4	64.8 ± 13.7	0.34	67.7 ± 13.5	64.4 ± 12.2	0.22
Sex (male/female)^b^	24/17	32/23	0.97	34/30	22/10	0.14	21/18	35/22	0.46	20/22	36/18	0.06
Localization^b^												
Tongue	12	19	0.59	17	14	0.27	9	22	0.11	15	16	0.46
Maxillary gingiva	8	7		13	2		7	8		7	8	
Mandibular gingiva	13	23		24	12		20	16		17	19	
Buccal mucosa	2	2		3	1		1	3		1	3	
Plantar of the mouth	6	4		7	3		2	8		2	8	
UICC stage^b^												
I	3	7	0.31	8	2	0 65	6	4	0.28	6	4	0.34
II	10	21		21	10		9	22		16	15	
III	8	8		9	7		6	10		5	11	
IV	20	19		26	13		18	21		15	24	

OSCC, oral squamous cell carcinoma; UICC, international union for cancer control; FAK, focal adhesion kinase; pFAK, phosphorylated FAK.

^a^ Student’s t-test.

^b^ Chi-squared test.

### Prognosis comparison between integrin-, FAK-, and pFAK-positive and negative groups

We compared cervical lymph node metastasis and OS rate between the positive and negative groups for each integrin-related molecule. Our univariate analysis indicated significantly more subjects with lymph node metastasis at the baseline in the integrin β8-positive compared to the negative group (Table 3, *p* < 0.05). We changed the applied variables for our multivariate logistic regression analysis (Table 4) and observed that the integrin β8 expression was independently associated with cervical lymph node metastasis (odds ratio 0.33, 95% CI 0.13–0.79, *p* < 0.05).

**TABLE 3:** Univariate analysis to determine associations with lymph node metastasis.

Category		Lymphonode metastases		*p*-value^a^
		No	Yes	
Age	<65/65≤	19/32	26/19	0.04*
Integrin αv	Negative/Positive	19/32	22/23	0.25
Integrin β1	Negative/Positive	36/15	28/17	0.39
Integrin β6	Negative/Positive	22/29	17/28	0.59
Integrin β8	Negative/Positive	29/22	13/32	0.01*
FAK	Negative/Positive	24/29	19/28	0.04*
pFAK	Negative/Positive	25/29	20/28	0.59

*p < 0.05 (statically significant).

FAK, focal adhesion kinase; pFAK, phosphorylated FAK.

^a^ Chi-squared test.

**TABLE 4:** Multivariate analysis to determine associations with lymph node metastasis.

Category		Reference	Odd ratio	95% CI		*p*-value^a^
				Lower	Upper	
Age	<65/65≤	65≤	2.24	0.95	5.41	0.06
Integrin αv	Negative/Positive					
Integrin β1	Negative/Positive					
Integrin β6	Negative/Positive					
Integrin β8	Negative/Positive	Positive	0.33	0.13	0.79	0.01*
FAK	Negative/Positive	Positive	0.47	0.17	1.19	0.11
pFAK	Negative/Positive					

*p < 0.05 (statically significant).

CI, confidence of interval; FAK, focal adhesion kinase; pFAK, phosphorylated FAK.

^a^ Logistic multivariate analysis.

**FIGURE 2:**
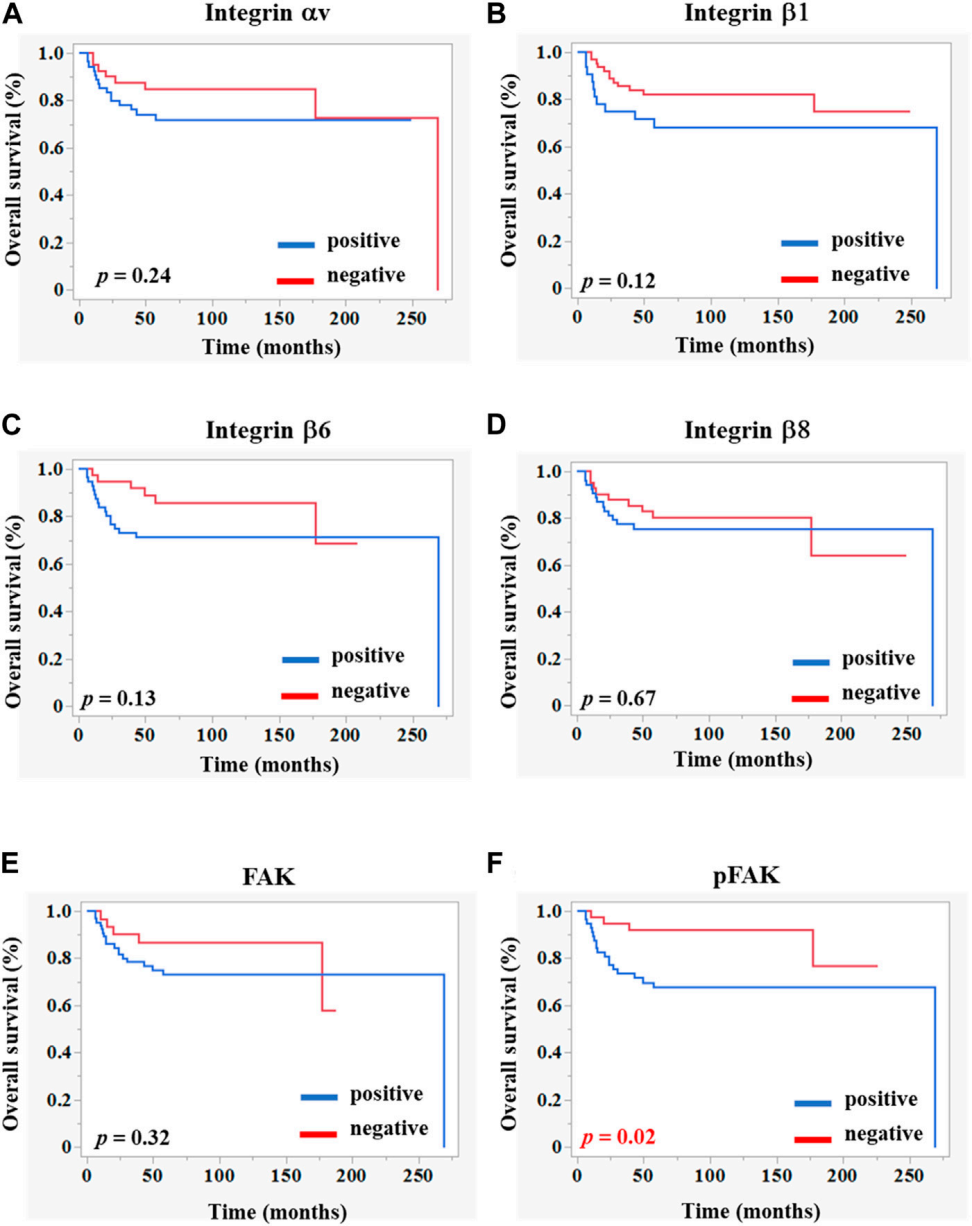
Kaplan–Meier curves of overall survivals according to the integrin-related protein expression in OSCC tissues. Each graph indicates the overall survival of patients with OSCC comprising positive (blue line) and negative (red line) groups of integrins **(A–D)**, FAK **(E)**, and pFAK **(F)**. The statistical differences were determined using the log-rank test. *p* < 0.05 (statically significant).

The OS rate was significantly lower in the pFAK expression–positive group than in the–negative group (Figure 2). We conducted Cox regression analyses incorporating an interaction term between age, stage, and immunostaining expression of each molecule and demonstrated a significant correlation between the hazard ratio of the pFAK-positive group (hazard ratio 0.35, 95% CI 0.14–0.87, *p* < 0.05) and the OS rate in our multivariate analysis (Table 5).

**TABLE 5:** Univariate and multivariate Cox regression analyses to determine associations with OS.

	Univariate analysis					Multivariate analysis				
Category	Reference	HR	95% CI		*p*-value^a^	Reference	HR	95% CI		*p*-value^a^
			Lower	Upper				Lower	Upper	
Age	65≤	1.1	0.51	2.32	0.82	65≤	0.77	0.36	1.66	0.5
Stage	Advance	0.24	0.09	0.64	0.004*	Advance	0.22	0.08	0.61	0.003*
Integrin α-V	Positive	0.84	0.39	1.83	0.67					
Integrin β1	Positive	0.57	0.27	1.21	0.14					
Integrin β6	Positive	0.48	0.2	1.14	0.1					
Integrin β8	Positive	0.75	0.34	1.65	0.48					
FAK	Positive	0.72	0.31	1.71	0.46					
pFAK	Positive	0.37	0.15	0.92	0.03*	Positive	0.35	0.14	0.87	0.02*

*p < 0.05 (statically significant).

^a^ Cox proportional hazards model.

HR, hazard ratio; CI, confidence of interval; FAK, focal adhesion kinase; pFAK, phosphorylated FAK.

### Relation between the expression of integrin family members, FAK and pFAK, and that of PD-1 and PD-L1 in OSCC

We examined the relation between the expression of integrin family members, FAK and pFAK, and that of PD-1 and PD-L1 in OSCC using immunostaining for PD-1 and PD-L1 (Supplementary Figure S1). However, no significant relationship was found between the expression levels of these molecules (Table 6). We also evaluated the OS rates based on PD-1 or PD-L1 expression in groups with or without the expressions of each inregrin-related molecule in OSCC; however, no significant differences were observed (Supplementary Figures S2, S3).

**TABLE 6:** Comparison of PD-1 or PD-L1 expression between positive and negative groups for integrin or FAK expression.

Category	PD-L1			PD-1		
	Positive	Negative	*p*-value^a^	Positive	Negative	*p*-value^a^
Integrin αv (positive/negative)	6/9	22/20	0.41	12/9	17/21	0.42
Integrin β1 (positive/negative)	3/12	11/31	0.63	3/18	11/27	0.19
Integrin β6 (positive/negative)	8/7	26/16	0.56	15/6	20/18	0.15
Integrin β8 (positive/negative)	9/6	23/19	0.73	13/8	21/17	0.62
FAK (positive/negative)	9/6	27/15	0.77	16/5	22/16	0.15
pFAK (positive/negative)	8/7	21/21	0.82	11/10	18/20	0.71

FAK, focal adhesion kinase; pFAK, phosphorylated FAK; PD-1, programmed cell death 1, PD-L1 PD-ligand 1.

^a^ Chi-squared test.

## Discussion

Integrins are heterodimeric plasma membrane proteins consisting of two transmembrane subunits (α- and β-chain). As ECM protein receptors, integrins regulate cell adhesion to the ECM and also transmit signals that regulate cell growth, differentiation, and motility [11–13]. Integrins are central to cancer invasion and metastasis as tumorigenesis, cancer cell motility, and proteolytic enzyme production regulators [46–48]. Therefore, we investigated whether integrins, FAK, or pFAK expression could serve as prognostic biomarkers for OSCC.

Multiple studies documented that integrin αV family members are closely associated with malignant tumor growth and progression [31–35]. Among them, integrin αVβ3 reportedly regulates proteolytic activity on cancer cell membranes as a plasma membrane receptor for active matrix metalloproteinase-2 (MMP-2), beyond acting as a cancer cell motility and promoter of angiogenic factor [49]. We have previously demonstrated that integrin αV is a plasma membrane receptor for active MMP-2 and that type I collagen activates FAK and MEK/ERK in OSCC cells via integrin αV, thereby increasing their proliferation [36, 50]. In this study, we examined the immunohistochemical expression of integrin αV and its counterpart integrins β1, β3, β5, β6, and β8 in resected tissue before treatment, then analyzed their association with clinicopathological factors, stage classification, and the OS rate. In addition, we examined FAK and pFAK expressions, involved in integrin-mediated signal transduction as well as malignant tumor growth and prognosis, in OSCC tissues and their association with the abovementioned clinical variables. Our results showed that integrin αV, β1, β6, and β8 were expressed in OSCC cells, but integrin β3 and β5 were not. To date, no studies reported integrin β3 or β5 protein expression in OSCC tissue. Kurokawa et al. reported that the integrin β5 mRNA is expressed in OSCC tissues [51]. Furthermore, it has been reported that the integrin β5 mRNA is also expressed in OSCC-derived cultured cells [52]. These discrepancies need to be examined in the future. We observed no statistically significant differences in gender, age, stage classification, or tissue differentiation between the integrin αV, β1, β6, and β8 positive and negative groups. Metastasis to the regional lymph nodes was significantly higher in the integrin β8-positive than that in the negative group, confirming our previous findings [36]. However, our comparison of the OS rate between the positive and negative groups for each integrin expression indicated no significant difference between the two groups.

Integrins bind to ECM proteins such as laminin and fibronectin in the extracellular domain, whereas the intracellular domain binds various signaling factors and actin-binding proteins directly or indirectly [15–17]. Integrins themselves do not exert any kinase activity, but when cells bind extracellular matrix proteins via integrins, FAK, an intracellular domain-binding protein of integrins, is activated, leading to mitogen- (MAP) kinase activation, thereby regulating cell proliferation as well as PI3-Akt pathway and Rho family small G protein activation, which regulate cell survival and cell motility and intracellular skeletal reorganization, respectively [18–22, 53]. We previously described that culturing integrin αV-overexpressing SCCKN cells transfected with the αV gene on type I collagen induced rapid FAK, MEK, and ERK1/2 phosphorylation compared to the control [36]. The OS rate in patients with OSCC was significantly lower in the pFAK-positive than in the negative group. No previous studies reported that integrin β8 or pFAK expression would play any role in the prognosis of patients with OSCC. To assess the predictive potential of integrins in immunotherapy, some studies have examined their association with immunological markers, including PD1, PD-L1, and TILs [54–57]. In gastric cancer, high expression levels of integrin β5 have been linked to poor prognosis and have been identified as an independent prognostic factor. Moreover, integrin β5 expression was positively correlated with the infiltration levels of CD4^+^ T cells, macrophages, and dendritic cells, particularly where lower macrophage infiltration led to improved prognosis in gastric cancer [54]. In patients with gastric cancer, integrin β1 and PD-L1 expression levels were correlated, as confirmed at the protein level via immunohistochemical analysis [57]. Subgroups with higher expression levels of integrin β4/PD-L1 and CD8/PD-1 in oral cancer exhibited a relatively better prognosis than other subgroups [56]. Therefore, we conducted IHC analysis to explore the correlation of integrin, FAK, and pFAK expression levels with PD-1 or PD-L1 expression. Although there was a trend toward a higher survival rate in the integrin- or FAK-negative group/PD-1- or PD-L1-positive group, this difference was not statistically significant.

This study has some limitations that must be considered. First, as it is a single-center, retrospective case-controlled study with a small sample size, regional bias might occur. Second, intratumor heterogeneity could affect integrin, FAK, and pFAK expression assessment. Third, basic factors such as smoking history, alcohol consumption history, and systemic diseases were not considered. Finally, because this was a retrospective study, the assessment of DOI for TNM classification (UICC 8th edition) at baseline was performed using histopathological specimens, which may differ from the assessment based on clinical findings.

Our results indicated significantly lower OS rates in pFAK-positive patients with OSCC compared to their negative counterparts, and cervical lymph node metastasis significantly increased in integrin β8-expressing patients with OSCC. Our results indicate that pFAK could be used as a potent prognostic factor for the OS rate and integrin β8 as a predictor of regional lymph node metastasis in patients with OSCC. We believe there is future potential for the development of new diagnostic and therapeutic methods for oral cancer targeting integrin β8 and pFAK.

## Data Availability

The original contributions presented in the study are included in the article/Supplementary Material, further inquiries can be directed to the corresponding authors.
