# Supplementary material for: Clinical significance of integrin αV and β superfamily members and focal adhesion kinase activity in oral squamous cell carcinoma: a retrospective observational study
Source: Pathol Oncol Res. 2024 Jan 18;30:1611571. doi: 10.3389/pore.2024.1611571 (PMC10830843; doi:10.3389/pore.2024.1611571)
Supplement: Supplementary file 1 [file DataSheet1.pdf]

Table S1 List of primary and secondary antibodies

	Primary antibody (Company)	Host	Dilution ratio	Antigen retrieval	Secondary antibody (Company)
Integrin αv	SC-6617R (Santa Cruz, Santa Cruz, CA, USA)	Rabbit	1/100	Citrate buffer	HRP-conjugated anti-rabbit IgG antibody (Agilent, Santa Clara, CA, USA)
Integrin β1	#9699 (Cell Signaling Technology, Danvers, MA, USA)	Rabbit	1/400	Citrate buffer	
Integrin β3	#13166 (Cell Signaling Technology)	Rabbit	1/500	Citrate buffer	
Integrin β5	SC-14010 (Santa Cruz)	Rabbit	1/200	Citrate buffer	
Integrin β6	19695-1-AP (Proteintech, Chicago, IL, USA)	Rabbit	1/200	none	
Integrin β8	SC-14010 (Santa Cruz)	Rabbit	1/200	none	
FAK	SC-558 (Santa Cruz)	Rabbit	1/200	EDTA	
pFAK	bs-3159 (Bioss Inc., Woburn, MA, USA)	Rabbit	1/1000	none	
PD-L1	17952-1-AP (Proteintech)	Rabbit	1/200	EDTA	
PD-1	#43248 (Cell Signaling Technology)	Mouse	1/200	Citrate buffer	HRP-conjugated anti-mouse IgG antibody (Agilent)

FAK focal adhesion kinase; pFAK phosphorylated FAK; PD-1 programmed cell death 1; PD-L1 programmed cell death ligand 1; EDTA ethylenediaminetetraacetic acid; HRP horseradish peroxidase

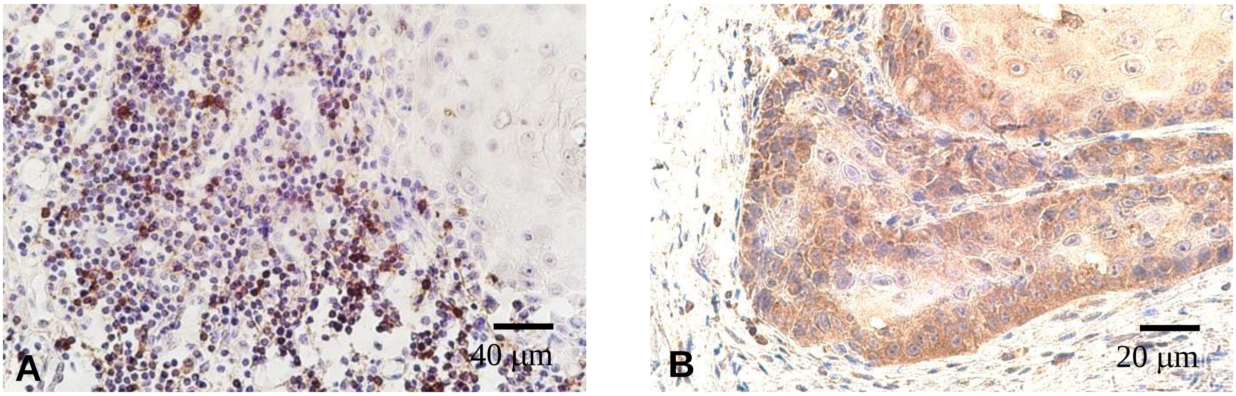

PD-1 **PD-L1**

Fig. S1. Immunohistochemical expression of PD-1 and PD-L1 in OSCC tissues. Both of PD-1 (A, original magnification ×100) and PD-L1 (B, original magnification ×200) were expressed in OSCC tissue.

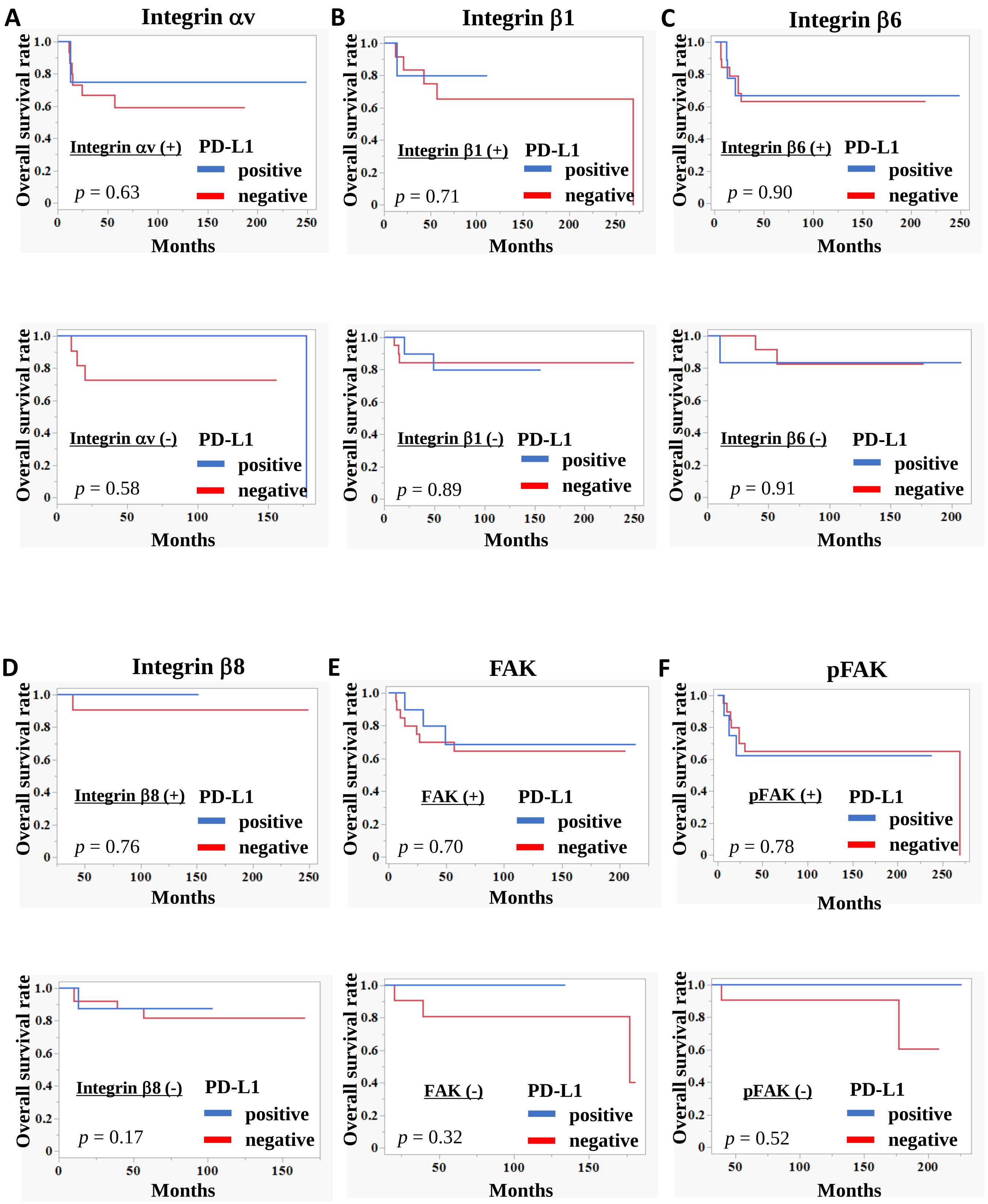

Fig. S2. Kaplan–Meier curves of overall survival based on PD-L1 expression in OSCC tissues with and without integrin-related protein expression. Graphs indicate the overall survival rate of patients with OSCC, comprising positive (blue line) and negative (red line) integrin (A-D), FAK (E), and pFAK (F) expression groups. Statistical differences were determined using the log-rank test.

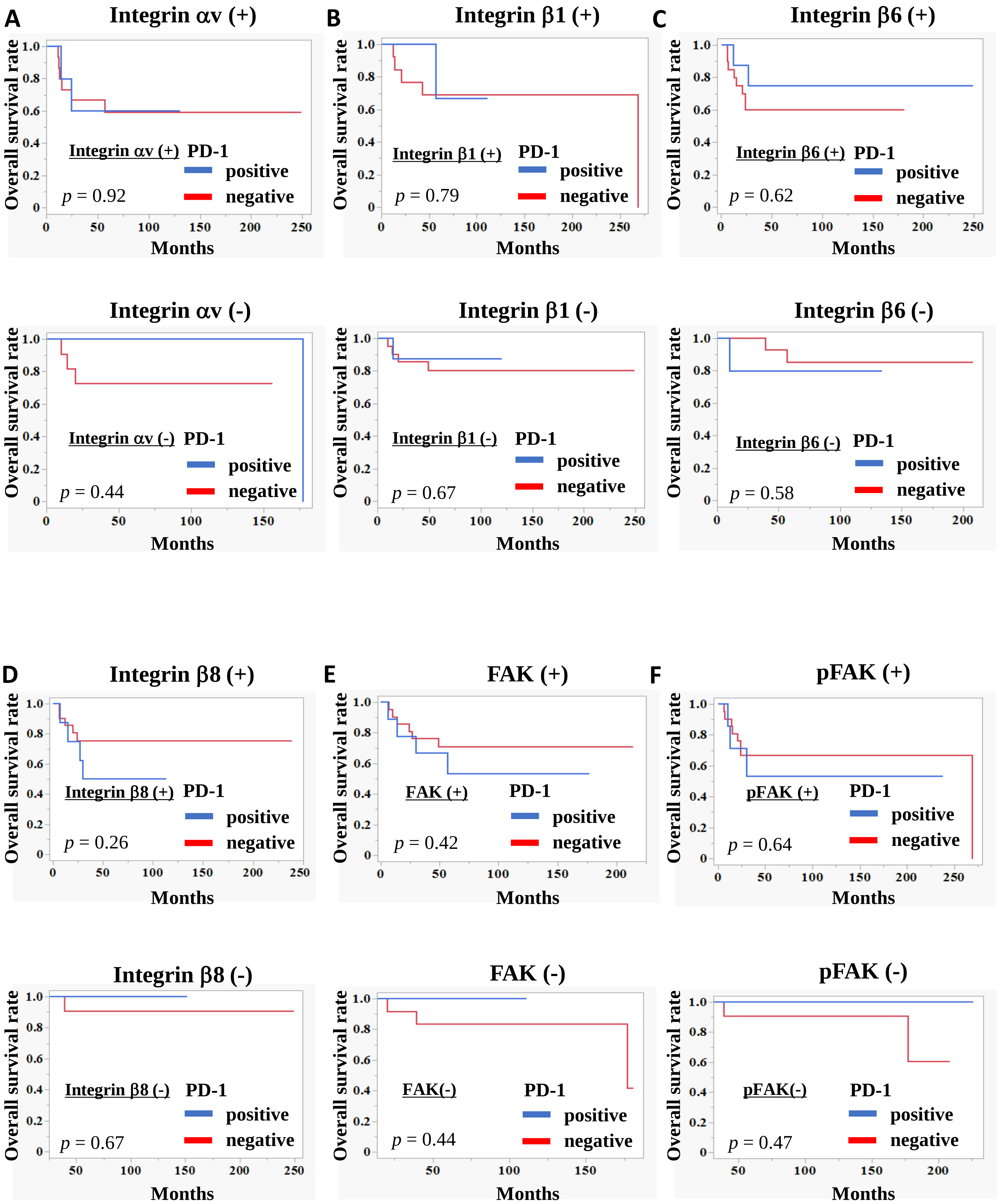

Fig. S3. Kaplan–Meier curves of overall survival based on PD-1 expression in OSCC tissues with and without integrin-related protein expression. Graphs indicate the overall survival rate of patients with OSCC, comprising positive (blue line) and negative (red line) integrin (A-D), FAK (E), and pFAK (F) expression groups. Statistical differences were determined using the log-rank test.
